# Use of Claims Data to Screen for Functional Limitations Among Medicare Beneficiaries

**DOI:** 10.1001/jamahealthforum.2026.1388

**Published:** 2026-06-12

**Authors:** Megan Mathews, Steven C. Martino, Denis Agniel, Helin G. Hernandez, Megan K. Beckett, Debra Saliba, Jacob W. Dembosky, Nathan Orr, Marc N. Elliott

**Affiliations:** 1 RAND, Santa Monica, California; 2 RAND, Pittsburgh, Pennsylvania; 3 Geriatric Research Education and Clinical Center, VA Medical Center-Los Angeles, Los Angeles, California; 4 Borun Center for Gerontological Research, University of California, Los Angeles

## Abstract

**Importance:**

The only functional impairment data available for all Medicare beneficiaries identify those who qualified for Medicare before age 65 years due to work-related disability. This administrative measure omits impairments arising after age 64 years or affecting nonwork-related activities. Surveys may offer more accurate assessments, but survey data on functional limitations are not routinely available. Claims data may provide a practical, scalable alternative.

**Objective:**

To describe the development of a survey-based index of functional limitations and a new claims-based model for predicting limitations.

**Design, Setting, and Participants:**

Data from respondents to the 2024 Medicare fee-for-service (FFS) Consumer Assessment of Healthcare Providers and Systems (CAHPS) survey were used to create the FFS CAHPS functional limitations index (FCFLI), a measure of the extent to which functional limitations affect overall health and the benchmark for the study’s claims-based measure. The FCFLI scores of respondents were linked to Medicare FFS claims from April 2023 to March 2024 to develop a claims-based predictive model (FCFLI-claims). The final model was applied to all FFS beneficiaries enrolled in Medicare Parts A and B during the same period.

**Main Outcomes and Measures:**

The FCFLI was derived from beneficiaries’ self-rated health and ability to perform 6 basic activities of daily living and 1 instrumental activities of daily living (collectively, I/ADLs). Nine sets of claims-based indicators of functional impairment were used to predict FCFLI scores.

**Results:**

Data were collected from 67 596 respondents (53.4% female; mean [SD] age, 75 [8] years). In the FCFLI model, all I/ADL indicators were negatively associated with self-rated health, with the largest effect estimates for difficulty walking (−13.9 points; 95% CI, −14.6 to −13.2 points) and running errands (−11.7 points; 95% CI, −12.7 to −10.8 points). In the FCFLI-claims model, 14 claims indicators had relative importance values that were 5% or greater of the maximum value. The model reliably identified beneficiaries with functional limitations (80.4% positive predictive value). Applied to the 2024 Medicare FFS population, approximately 12% were identified as likely to have functional limitations. Nearly 63% (approximately 2 450 000) of those identified by the algorithm were not identified by Centers for Medicare & Medicaid Services administrative measure of disability, which focuses on limitations that develop before age 65 years.

**Conclusions and Relevance:**

In this cross-sectional study, FCFLI-claims identified beneficiaries likely to have functional limitations who could be prioritized for a survey assessment to verify the algorithm’s results. Integrating this information with entitlement data could enhance Centers for Medicare & Medicaid Services’ monitoring of functional status, especially among age-eligible beneficiaries.

## Key Points

**Question:**

Can Medicare claims data be used to identify beneficiaries with a high likelihood of having functional limitations?

**Findings:**

In this cross-sectional study of data from 67 596 survey respondents, an algorithm based on a parsimonious set of claims indicators achieved a positive predictive value of 80% in identifying Medicare fee-for-service beneficiaries who were functionally limited. Nearly 63% of those identified by the algorithm were not identified as functionally limited in Centers for Medicare & Medicaid Services administrative data, which focuses on limitations that arise before age 65 years.

**Meaning:**

These finding suggest this claims-based algorithm may serve as an effective screening tool to identify beneficiaries who should be prioritized for a survey assessment to verify the results of the algorithm.

## Teaser

This cross-sectional study assesses the usefulness of claims-based data to identify Medicare beneficiaries with functional limitations.

## Introduction

Approximately 1 in 10 US adults have functional limitations that substantially restrict 1 or more major life activities.^1^ These individuals often face systemic barriers to health care, including physical inaccessibility and communication challenges,^1,2,3^ and are more likely to report poor health and develop preventable chronic conditions.^4,5,6^

Accurate data are essential for identifying and addressing the needs of people with functional limitations. Among Medicare beneficiaries, the only functional status information currently available for all enrollees is whether they originally qualified for Medicare before age 65 years due to Social Security Disability Insurance eligibility. However, this criterion is limited because it captures only disabilities that affect work and arise before age 65 years, identifies people who may have different health care use than those who develop limitations later,^7^ and lacks detail on severity. Consequently, it likely misses many beneficiaries with functional impairments.

Survey-based measures remain the standard for assessing functional limitations. For instance, the Functional Limitations Index developed by Mathews et al^8^ summarizes self-reported limitations across 5 domains—vision, hearing, cognition, mobility, and self-care—capturing both severity and association with overall health. Despite its advantages, this and other survey-based approaches may be costly to implement broadly.

Claims or administrative data offer an alternative for identifying individuals with probable functional limitations because they are available for all Medicare beneficiaries.^9^ One example is the Access Risk Classification System (ARCS), which assesses functional status based on indicators of access to routine care.^10^ Another example is an algorithm developed by Ben-Shalom and Stapleton,^11^ validated using 2003 to 2006 Medicare Current Beneficiary Survey data. Their model incorporated indicators from ARCS, the Chronic Illness and Disability Payment System (CDPS),^12^ the Social Security Administration Health Information Technology (SSA-HIT) business rules,^13^ and Medicare claims related to psychiatric, cognitive, and neurodevelopmental disorders to predict disability.

However, claims-based approaches often fail to identify many beneficiaries with functional limitations. This may occur because some individuals generate few claims, because longer claims histories are needed to reveal patterns, or because claims data cannot fully capture aspects such as severity. These models may also misclassify individuals without impairments as functionally limited, ie, have low positive predictive value, when short-term health events mimic chronic impairment.

Although claims data cannot identify all limitations, a reasonably well-calibrated claims-based measure could efficiently flag individuals likely to have functional limitations, enabling targeted follow-up through survey-based assessment. Such a 2-step approach could identify beneficiaries overlooked by current entitlement-based indicators, enhancing the ability of the Centers for Medicare & Medicaid Services (CMS) and researchers to understand trajectories and needs for this population, informing program design and implementation.

Our objective was to develop a new claims-based model that predicts functional limitations among Medicare beneficiaries with high accuracy. To do so, we first needed a valid survey-based criterion that could be linked to claims data for a large sample. Although the Functional Limitations Index^8^ has strong validity, it is derived from items included in the National Health Interview Survey (NHIS), which is completed by relatively few Medicare beneficiaries. To overcome this limitation, we constructed a similar index using self-reported data on difficulty performing basic activities of daily living (ADLs) and the more complex task of running errands from the fee-for-service (FFS) Consumer Assessment of Healthcare Providers and Systems (CAHPS) survey, which is completed by more beneficiaries than the NHIS and may therefore have more robust statistical properties.

In this study, we describe the development of this new survey-based index of functional limitations, the FFS CAHPS Functional Limitations Index (FCFLI), and a new claims-based model for predicting it (FCFLI-claims model).

## Methods

This cross-sectional study follows the Strengthening the Reporting of Observational Studies in Epidemiology (STROBE) reporting guideline for cross-sectional studies. It was reviewed and approved by the RAND Human Subjects Protection Committee. Informed consent was waived due to use of deidentified data. We used the relevant portions of the Transparent Reporting of a Multivariable Prediction Model for Individual Prognosis or Diagnosis (TRIPOD) reporting guideline in reporting the results of our prediction modeling.

Our target population for this cross-sectional study was all Medicare FFS enrollees (N = 31 539 848). In the first phase of our analysis, we used data from 67 596 adult respondents to the 2024 Medicare FFS CAHPS survey^14^ (28% response rate)^15^ to develop the survey-based FCFLI. We excluded 4061 respondents who did not complete the 7 activity items or rate their health status, as both are required to compute the FCFLI.

Next, we merged FCFLI scores with Medicare FFS claims data via the CMS Integrated Data Repository^16^ to create the FCFLI-claims model, a claims-based model for predicting FCFLI. To align with the CAHPS 2024 fielding period (January through March 2024), we used claims data from April 2023 through March 2024, establishing a 1-year lookback period. To ensure complete claims data across all care settings, we excluded 5991 respondents enrolled in Medicare Advantage (Part C) or not continuously enrolled in both Parts A and B during the lookback period. This yielded a final analytic sample of 57 544 respondents with linked survey and claims data for model development. To evaluate the FCFLI-claims model, we used data from the 2023 CAHPS survey and linked claims files (eMethods in Supplement 1). We then applied the final FCFLI-claims model to estimate functional limitation status for all beneficiaries enrolled in Parts A and B, but not C, at any point between April 2023 and March 2024.

### Statistical Analysis

#### Analysis Phase 1: Creating the FCFLI

Construction of the FCFLI required assigning numeric values to the self-reported severity levels for each of the 6 ADL items and 1 instrumental activity of daily living (collectively, I/ADL) item in the FFS CAHPS survey, allowing us to combine these responses into a single, interpretable summary measure of functional limitation. Following Mathews et al,^8^ we assigned these values by calibrating responses to the I/ADL items against self-rated health. The underlying idea is that a limitation with a higher calibration with poorer self-rated health should receive a higher numeric value than one with a lower calibration. This use of self-rated health is purely instrumental; it serves as a means to construct a coherent functional limitation score, not as a primary outcome.

To quantify the relative effect estimates of different functional limitations on self-rated health, we used responses to the seven I/ADL items to estimate respondent self-rated health, linearly transformed to a 0 to 100 scale (from the original 5-point scale, 1 = poor to 5 = excellent) for interpretability. These seven I/ADL items assessed difficulty or inability in bathing, dressing, eating, transferring (getting in and out of a chair), walking, toileting, and running errands alone due to a physical, mental, or emotional condition. We also included as explanatory variables counts of the number of ADLs beyond 1 that a person had difficulty doing and of the number beyond 1 that a person was unable to do, as well as sociodemographic covariates (age, educational attainment, receipt of a Part D Low-Income Subsidy [LIS], and census division) known to be associated with self-rated health. Missing covariate values were imputed using the mean of nonmissing cases within the same state; overall, missingness was minimal (<2% for all covariates).

FCFLI scores were calculated as covariate-adjusted estimated health values using recycled predictions derived from each respondent’s reported functional limitations and assuming mean FFS population characteristics. These estimated values were then standardized (mean [SD] of 0 [1]). Covariate adjustment reduced response bias and mitigated differences associated with sociodemographic characteristics. All analyses incorporated CAHPS survey respondent weights to account for sample design, nonresponse, and poststratification adjustments.^17^ The eMethods in Supplement 1 contains additional detail on the development of the FCFLI, including approaches that were considered but ultimately not used.

#### Analysis Phase 2: Predicting FCFLI Scores From Claims Data

We used 9 groups of claims-based indicators to predict FCFLI scores: a 4-category ARCS,^10^ 20 CDPS^12^ indicators, 10 indicators from the Medicare Chronic Conditions Warehouse (CCW),^18^ SSA-HIT,^13^ Hierarchical Condition Category (HCC) risk scores,^19^ and the Quan-Charlson Comorbidity Index (QCCI).^20^ We excluded psychiatric, cognitive, and intellectual disability indicators from the SSA-HIT due to overlap with CDPS and CCW measures. Each index captures different dimensions of health status that could plausibly relate to functional limitations. ARCS reflects the need for care coordination and system accommodations; CDPS and HCC quantify diagnostic and cost-related risk; SSA-HIT identifies individuals with severe or disabling conditions; and the QCCI measures overall comorbidity burden. We also incorporated the Medicaid Rx model,^21^ total coverage days, and hospital readmissions to capture high health care use, common among adults with functional limitations, and to identify those at increased risk of such use (eTables 1-3 in Supplement 1).

For indicators with mutually exclusive categories, the no-risk or no-condition group served as the reference. We included administrative covariates (age, sex, dually eligible or LIS recipient) based on prior evidence of their relevance to predictive accuracy. To account for differences in data availability, we added variables for number of FFS-enrolled months, continuous Part D enrollment, and institutionalization during the lookback period. Missing covariate values were imputed using the mean of nonmissing cases within each state; overall, missingness was minimal (<1% of all covariates).

Claims-based predictors were constructed using both a 1-year (April 2023-March 2024) and a 2-year (April 2022-March 2024) lookback period to test whether extending the claims history improved prediction accuracy. We evaluated standard linear regression and a random forest algorithm to predict FCFLI scores from the claims-based indices and covariates (eMethods in Supplement 1). Predicted FCFLI-claims model scores were standardized (mean [SD] of 0 [1]), and CAHPS respondent weights were applied.

Model performance was evaluated using the root mean squared error (RMSE) to quantify the mean deviation between FCFLI-claims model predictions and observed FCFLI scores. The RMSE formula is available in the eMethods in Supplement 1. Lower RMSE values indicate better model fit, with 0 representing perfect prediction. RMSE was computed for both in sample (2024) and out of sample (2023) data to examine temporal generalizability. Less than 2% of respondents to the 2024 CAHPS survey were sampled for the 2023 survey, effectively making it an independent sample. We chose this evaluation strategy because it mirrors the intended use of the FCFLI-claims model: to be applied to these data in this context at different points.

After selecting the model with the lowest RMSE, we evaluated classification accuracy using positive predictive value (PPV). We chose PPV because the primary purpose of the FCFLI-claims model is to identify beneficiaries highly likely to have functional limitations, a subset that may be ideal for survey or other follow-up. Since follow-up has real costs, PPV provides a direct measure of the expected return on that investment.

PPV measured how accurately FCFLI-claims model scores identified individuals across 3 limitations groups: least, somewhat, and most limited. This categorization reflects the intended use of the FCFLI-claims model to distinguish beneficiaries by functional status. To define these groups, we evaluated combinations of cutpoints dividing the FCFLI and FCFLI-claims model score distributions into least, somewhat, and most limited groups, and searched for a combination that maximized PPVs. We also examined PPV for a binary classification: least limited vs at least somewhat limited. Although a PPV of 80% is often cited as a benchmark for strong performance,^22,23^ lower PPVs may be acceptable when identifying low-prevalence subgroups.

We applied the final FCFLI-claims model to the total 2024 FFS population, using selected model specifications and cutpoints to assign estimated scores and categorize beneficiaries for comparison with CMS administrative classifications based on the original reason for Medicare entitlement.

SAS version 9.4 (SAS Institute Inc) was used for all analyses except the random forest modeling, which was performed using R version 4.4.1 (R Foundation for Statistical Computing). All statistical tests were 2-sided, and a *P* < .05 was considered statistically significant.

## Results

### Comparison of the Full Medicare FFS Population With 2024 FFS CAHPS Respondents

Compared with the total Medicare FFS population, the 63 535 (46.5% male; 53.5% female; mean [SD] age, 76 [11] years) 2024 FFS CAHPS respondents whose data were used in constructing the FCFLI were older (aged 70 years and older: 74.8% vs 67.4%), less likely to be dually eligible for Medicaid or receive the LIS (12.3% vs 19.3%), and more likely to have been continually enrolled in FFS during the 1-year lookback period (86.2% vs 80.1%) (Table 1).

**Table 1.** Characteristics of the Total 2024 Medicare FFS Population and Those Included in Survey-Based and Claims-Based Measurement Models

Characteristic	Mean % (95% CI)		
	All Medicare FFS enrollees (N = 31 539 848)	Difference from total population^a^	
		Included in FCFLI analysis (n = 63 535)	Included in FCFLI-claims model analysis (n = 57 544)
Sex			
Female	54.2 (54.2 to 54.3)	−0.7 (−0.8 to 6.6)	−1.1 (−9.2 to 6.9)
Male	45.8 (45.7 to 45.8)	0.7 (−6.6 to 8.0)	1.1 (−6.9 to 9.2)
Age, y			
18 to 64	10.2 (10.2 to 10.3)	−3.1 (−7.1 to 0.9)	−2.3 (−6.7 to 2.1)
65 to 69	22.3 (22.3 to 22.4)	−4.3 (−10.3 to 1.6)	−2.3 (−8.8 to 4.1)
70 to 75	23.3 (23.2 to 23.3)	0.9 (−5.7 to 7.5)	3.6 (−3.6 to 10.8)
76 to 79	18.8 (18.8 to 18.8)	9.8 (2.8 to 16.8)^b^	1.9 (−4.7 to 8.4)
80 to 84	12.2 (12.2 to 12.2)	−0.8 (−5.7 to 4.2)	0.5 (−4.9 to 5.9)
≥85	13.1 (13.1 to 13.1)	−2.5 (−7.3 to 2.3)	−1.3 (−6.5 to 3.9)
Dually eligible for Medicare and Medicaid or LIS recipient	19.3 (19.3 to 19.3)	−7.0 (−11.7 to −2.2)^b^	−7.5 (−12.7 to −2.3)^b^
Original reason for Medicare entitlement: disability	18.5 (18.4 to 18.5)	−2.7 (−8.0 to 2.5)	−3.2 (−9 to 2.6)
Continuous Part D enrollment^c^	62.4 (62.4 to 62.4)	−5.0 (−12.6 to 2.7)	1.4 (−6.4 to 9.2)
Continuous FFS enrollment^c^	80.1 (80.1 to 80.1)	6.1 (0.8 to 11.4)^b^	15.7 (12.5 to 19.0)^b^
Census division			
New England	5.4 (5.4 to 5.4)	−0.7 (−3.9 to 2.6)	−0.1 (−3.7 to 3.5)
Mid-Atlantic	12.4 (12.4 to 12.4)	−0.9 (−5.9 to 4.0)	0.3 (−5.1 to 5.7)
East North Central	13.8 (13.8 to 13.8)	−1.8 (−6.8 to 3.3)	−0.4 (−5.9 to 5.1)
West North Central	7.2 (7.1 to 7.2)	−0.9 (−4.6 to 2.9)	−0.2 (−4.3 to 3.9)
South Atlantic	21.1 (21.1 to 21.1)	−1.9 (−8.0 to 4.2)	0.3 (−6.4 to 6.9)
East South Central	5.9 (5.9 to 5.9)	−0.7 (−4.2 to 2.7)	−0.2 (−3.9 to 3.6)
West South Central	10.2 (10.2 to 10.2)	−1.0 (−5.5 to 3.5)	0.0 (−4.9 to 4.9)
Mountain	7.2 (7.2 to 7.2)	−0.3 (−4.2 to 3.6)	0.5 (−3.8 to 4.8)
Pacific	13.9 (13.8 to 13.9)	−1.4 (−6.5 to 3.7)	0.0 (−5.6 to 5.5)
Missing	2.7 (2.7 to 2.7)	9.7 (4.6 to 14.8)^b^	−0.1 (−2.7 to 2.5)

Abbreviations: FCFLI, Fee-for-Service Consumer Assessment of Healthcare Providers and Systems (CAHPS) Functional Limitation Index; FFS, fee-for-service; LIS, low-income subsidy.

^a^ Those included in the FCFLI analyses responded to the 2024 FFS CAHPS survey. Those included in the FCFLI-claims model analysis responded to the 2024 FFS CAHPS survey and had linked claims data during the 1-year lookback period. Percentages shown in these 2 columns were adjusted using FFS CAHPS respondent weights.

^b^ Difference of 5% or more.

^c^ During the 1-year claims lookback period.

### Endorsement of IADL Limitations on the FFS CAHPS Survey

Approximately 30% of FFS CAHPS respondents reported some level of difficulty—either difficulty performing or complete inability—with at least one I/ADL. The most commonly reported limitation was walking (24.9%), followed by transferring (17.5%), running errands alone (15.6%), bathing (13.0%), dressing (11.4%), using the toilet (8.7%), and eating (6.1%). Approximately 3% to 4% of respondents reported being unable to perform each activity (eTable 4 in Supplement 1).

### Prediction of Self-Rated Health From Self-Reported Limitations to Create the FCFLI

All I/ADL indicators were significantly and negatively associated with self-rated health (Table 2; eTable 5 in Supplement 1). Limitations were associated with mean point decreases in self-rated health for each increase in level of severity: walking, −13.9 (95% CI, −14.6 to −13.2; *P < *.001), running errands, −11.7 (95% CI, −12.7 to −10.8; *P < *.001), transferring, −9.0 (95% CI, −10.0 to −8.0; *P < *.001), eating, −6.7 (95% CI, −8.1 to −5.2; *P < *.001), bathing, −6.3 (95% CI, −7.5 to −5.1; *P < *.001), dressing, −6.0 (95% CI, −7.4 to −4.5; *P < *.001), and using the toilet, −3.3 (95% CI, −4.8 to −1.8; *P < *.001). Positive coefficients on the 2 count variables indicated diminishing marginal effect estimates with additional limitations. The model estimating self-rated health showed stable performance across years (RMSE 20.9 in sample, 21.1 out of sample).

**Table 2.** Final Multivariate Regression Model Estimating Self-Rated Health From Limitations With Basic or Instrumental Activities of Daily Living, 2024 Medicare Fee-for-Service Consumer Assessment of Healthcare Providers and Systems Survey

Limitation	Coefficient (95% CI)^a^	*P* value
Limitation, per unit^b^		
Bathing	−6.3 (−7.5 to −5.1)	<.001
Dressing	−6.0 (−7.4 to −4.5)	<.001
Eating	−6.7 (−8.1 to −5.2)	<.001
Transferring (getting in and out of a chair)	−9.0 (−10.0 to −8.0)	<.001
Walking	−13.9 (−14.6 to −13.2)	<.001
Using the toilet	−3.3 (−4.8 to −1.8)	<.001
Difficulty running errands alone (0 = no difficulty, 1 = has difficulty)	−11.7 (−12.7 to −10.8)	<.001
Count of limitations beyond 1, by severity level		
Have difficulty (per 0-6 additional limitations)	3.9 (3.1 to 4.7)	<.001
Unable to do (per 0-5 additional limitations)	16.6 (14.9 to 18.2)	<.001

^a^ Cell entries are coefficients and associated 95% CIs from a linear regression model estimating self-rated health scored 0 to 100, where 0 represents poor health and 100 represents excellent health. N = 63 535. Adjusted R^2^ for model: 0.257. Root mean square error: 21.0. Additional covariates were included in this model; full model results can be found in eTable 5 in Supplement 1.

^b^ Units of limitation: 0 = no difficulty, 1 = has difficulty, 2 = unable to do.

### Selection of a Preferred FCFLI-Claims Modeling Approach

**Figure 1.**
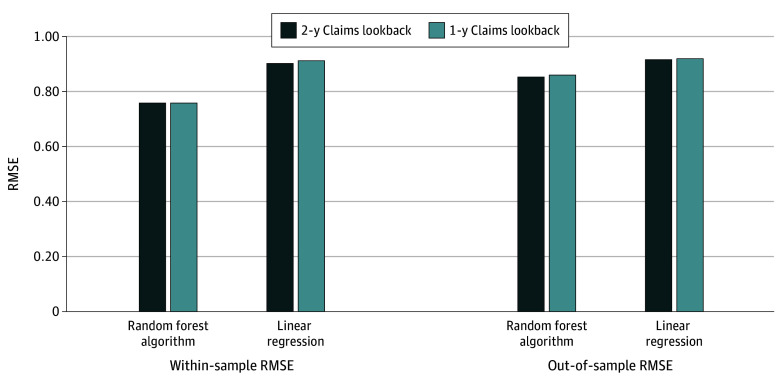
Bar Graph of the Root Mean Squared Error (RMSE) for Each Medicare Fee-for-Service Consumer Assessment of Healthcare Providers and Systems Functional Limitations Index Claims Model The RMSE formula is available in the eMethods of Supplement 1.

Distributions of the claims-based indictors included in models predicting FCFLI scores are shown in eTables 6 and 7 in Supplement 1. We hypothesized that extending the claims lookback period from 1 to 2 years would improve predictive accuracy by capturing additional services used by highly impaired beneficiaries who use care less often. However, RMSEs were similar for the 1-year and 2-year lookback periods both within sample and out of sample across modeling approaches (Figure 1), indicating that extending the lookback period was not associated with improved prediction accuracy. Given comparable performance and greater processing efficiency, we selected the 1-year lookback for subsequent modeling. With this approach, RMSEs were 0.91 (within sample) and 0.92 (out of sample) for the linear model and 0.76 and 0.84, respectively, for the random forest model. Given its lower RMSEs, we selected the random forest as the final modeling approach.

**Figure 2.**
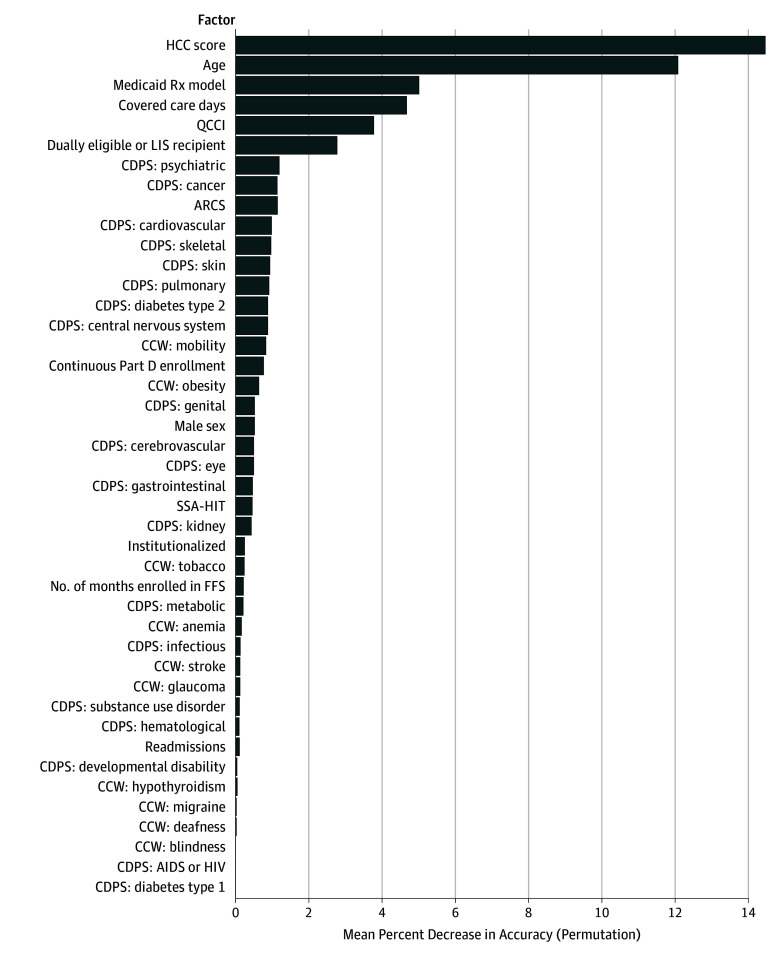
Bar Graph of the Relative Importance of Claims-Based Predictors in the Random Forest Model Predicting 2024 Survey-Based Fee-for-Service Consumer Assessment of Healthcare Providers and Systems Functional Limitation Index Scores (1-Year Lookback) Predictor importance was assessed using the random forest permutation method (mean decrease in accuracy), defined as the reduction in model performance when a predictor’s values are randomly permuted while all other variables remain unchanged. ARCS indicates access risk classification system; CCW, Chronic Conditions Warehouse; CDPS, Chronic Illness and Disability Payment System; FFS, fee for service; HCC, Hierarchical Condition Category; LIS, Low-Income Subsidy; QCCI, Quan-Charlson Comorbidity Index; SSA-HIT, Social Security Administration Health Information Technology.

Fourteen claims indicators had relative importance values 5% or greater of the maximum (ie, ≥0.72%) in predicting FCFLI scores, with these 7 explaining approximately one-quarter of the model’s total importance: HCC risk (14.4%), Medicaid Rx risk (5.0%), covered care days (4.7%), QCCI (3.8%), CDPS psychiatric (1.2%), CDPS cancer (1.1%), and ARCS risk (1.1%; Figure 2). Details on the linear regression model results are in eTable 8 in Supplement 1.

### Identification of Optimal Cutpoints for Determining Functioning Groups

FCFLI scores ranged from −4.49 to 0.95 (mean [SD], 0 [1.00]), and FCFLI-claims model scores ranged from −2.33 to 0.57 (mean [SD], −0.01 [0.53]). We tested 1269 combinations of cutpoints for defining the least, somewhat, and most limited functioning groups (eTable 9 in Supplement 1), but none achieved acceptable PPVs for all 3 groups. Optimizing predictive accuracy for 1 group typically reduced accuracy for others (eFigure in Supplement 1).

Using a single −1.00 cutpoint to define a binary at least somewhat limited group (combining the somewhat and most limited categories) yielded optimal PPVs for both this group (80.4%) and the least limited group (87.1%). These findings indicate that the FCFLI-claims model can reliably identify beneficiaries with a high likelihood of functional limitations but is less effective at distinguishing among levels of severity.

### Application of the FCFLI-Claims Model Method to the Full FFS Population

Applying the final FCFLI-claims model and a −1.00 cutpoint identified approximately 12% (N = 3 896 246) of 2024 Medicare FFS beneficiaries as likely to have functional limitations. Compared with those unlikely to have functional limitations, these beneficiaries were more likely to have originally qualified for Medicare based on disability (37.1% vs 15.8%), to be dually eligible for Medicare and Medicaid or LIS recipients (50.6% vs 14.9%), to be female (59.1% vs 53.7%), and to be in either the younger age group (18-64 years) or older age group (≥85 years) (21.1% vs 8.7% and 38.3% vs 9.6%, respectively) (Table 3).

**Table 3.** Distribution of 2024 Fee-for-Service Medicare Enrollees Demographic Characteristics by FCFLI-Claims Model Predicted Probability Groupings

Characteristic	Likelihood of functional limitations, %	
	Less likely (FCFLI-claims model score greater than −1.00) (n = 27 633 602)	More likely (FCFLI-claims model score −1.00 or less) (n = 3 896 246)
All Medicare FFS enrollees	87.6	12.4
Original reason for Medicare entitlement		
Disabled	15.8	37.1
Not disabled	84.2	62.9
Dual eligibility/LIS recipient status		
Either dually eligible or an LIS recipient	14.9	50.6
Neither dually eligible nor an LIS recipient	85.1	49.4
Age, y		
18-64	8.7	21.2
65-69	24.5	7.5
70-74	25.2	9.5
75-79	19.9	11.2
80-84	12.2	12.3
≥85	9.6	38.3
Sex		
Male	46.3	40.9
Female	53.7	59.1

Abbreviations: FCFLI, Fee-for-Service CAHPS Functional Limitation Index; FFS, fee-for-service; LIS, low-income subsidy.

Additionally, 62.8% of beneficiaries identified by the model as likely to have functional limitations (N = 2 450 385) were not identified as disabled by the CMS administrative measure of disability, the original reason for entitlement code. This subgroup tended to be older, less likely to be dually eligible or LIS recipients, and slightly more likely to be female than beneficiaries identified as disabled by the original reason for entitlement code but also identified by the FCFLI-claims model (eTable 10 in Supplement 1).

## Discussion

Similar to the survey-based measure developed by Mathews et al,^8^ the FCFLI is a valid interpretable measure of functioning that is independent of sociodemographic characteristics and health conditions. It can be used by CMS and others to monitor care and outcomes among FFS beneficiaries with varying degrees of functional limitation. Similar to the FLI, the FCFLI summarizes the combined associations of multiple limitations with self-rated health using empirically derived weights and a term that accounts for the diminishing marginal impact of additional limitations. The FCFLI has 2 key advantages over the FLI: it explicitly includes cognitive and mental functioning and can be constructed for any Medicare beneficiary completing the FFS CAHPS survey, which covers far more beneficiaries than the NHIS on which the FLI is based.

Although the FFS CAHPS survey reaches many more beneficiaries than the NHIS, it represents only a fraction of the total FFS population. To address this, we developed the FCFLI-claims model, a model that predicts FCFLI scores using claims data. Despite the challenges of inferring functioning from claims, the FCFLI-claims model performed well in identifying beneficiaries likely to have functional limitations: 80% of those identified as at least somewhat limited were at least somewhat limited according to their FCFLI scores. This predictive accuracy exceeds that of prior claims-based models; for example, Ben-Shalom and Stapleton reported PPVs of 42.6% for adults aged 65 years or older and 56.3% for those aged 18 to 64 years.^11^

Applied to the full FFS population, the FCFLI-claims model identifies 12% of beneficiaries (approximately 3.9 million people) as having a high likelihood of being at least somewhat functionally limited. Compared with those with a low predicted probability, these beneficiaries were more often originally entitled due to disability, dually eligible for Medicaid or LIS recipients, aged 18 to 64 years or 85 years or older, and female. These patterns align with prior research,^24,25^ supporting the FCFLI-claims model construct validity. Notably, approximately 63% of those predicted as likely to be at least somewhat limited—nearly 2.5 million people—were not originally entitled via disability, underscoring the model’s utility in identifying unrecognized functional limitations.

Because claims data imperfectly capture functional limitations, they should complement—not replace—surveys. We recommend using the FCFLI-claims model as a screening tool to identify FFS beneficiaries likely to have limitations. For example, CMS could run the algorithm annually (or at another interval) to identify those with a high predicted probability of functional limitation and send them a brief follow-up survey containing I/ADL items to calculate FCFLI scores. Integrating these survey data with existing entitlement information could help CMS better quantify this population, monitor their health and care, and inform resource allocation for programs such as home-based and community-based services and long-term services and support. Because some people with functional limitations will be classified outside the high-probability group, we also recommend surveying a small sample of that group to ensure broader representation.

### Limitations

This study has limitations. As with other claims-based measures,^10,11^ the FCFLI-claims model was less effective in differentiating among levels of impairment. Beneficiaries with little or no impairment are easier to classify due to low health care use and few chronic conditions, whereas individuals with the greatest impairments may have limited access to care,^26,27^ resulting in sparse claims and potential misclassification. Some underestimation likely stems from missing Medicaid claims, which are not captured in Medicare data; including them could reduce prediction error, especially among beneficiaries originally entitled via disability. Our analysis is based on the Medicare FFS population and may not generalize to Medicare Advantage enrollees. Further, our model validation did not address transportability to different datasets, and performance may differ for other use cases. Moreover, the sample of survey respondents used to validate the FCFLI-claims model differed from the full Medicare FFS population in expected ways.^28^ We used nonresponse weights to minimize the effects of those differences on our results.

## Conclusions

In this cross-sectional study, FCFLI-claims model identified beneficiaries likely to have functional limitations who could be prioritized for a survey assessment to verify the algorithm’s results. Integrating this information with entitlement data could enhance Centers for Medicare & Medicaid Services’ monitoring of functional status, especially among age-eligible beneficiaries.

## Notes

Supplement 1. **eMethods.** Inclusion Criteria for Validation Sample **eTable 1.** Access Risk Classification System and Chronic Conditions Warehouse Claims-Based Indicators of Functional Status **eTable 2.** Chronic Illness and Disability Payment System (CDPS) Claims-Based Indicators of Functional Status **eTable 3.** Claims-Based Indicators of Functional Status and Indices Not in the Access Risk Classification System, Chronic Conditions Warehouse, or Chronic Illness and Disability Payment System **eTable 4.** Weighted Endorsement Rates of (Instrumental) Activities of Daily Living Among Eligible 2024 FFS CAHPS Survey Respondents (N = 63 535) **eTable 5.** Survey-Based FCFLI Linear Regression Model Coefficients **eTable 6.** Overall Means and Confidence Intervals for Binary Claims-Based Indicators of Functional Status for the Full Medicare Fee-for-Service Population and FCFLI-Claims Analytic Set **eTable 7.** Continuous Claims-based Indicators of Functional Status, Overall Means and Confidence Intervals for the Full Medicare Fee-for-Service Population and FCFLI-Claims Analytic Set **eTable 8.** Linear Model Results Predicting 2024 Survey-Based FCFLI Scores from Claims Variables (1-Year Lookback) **eTable 9.** Best Cut Points for Creating Functioning Groups Based on Positive Predicted Value (PPV) **eTable 10.** Distribution of 2024 Fee-for-Service Medicare Enrollees At Least Somewhat Functionally Limited by the FCFLI-Claims (score≤−1.00) Demographic Characteristics by Disability Insurance Status Original Reason for Medicare Entitlement **eFigure 1.** Positive Predictive Value (PPV) and Limitation Group Cut Point Optimization

Supplement 2.
Data Sharing Statement

## References

[1] National Center for Health Statistics. Health, United States: Functional Limitation. Accessed October 28, 2025. https://www.cdc.gov/nchs/hus/topics/functional-limitation.htm

[2] Iezzoni LI, Rao SR, Ressalam J, . Physicians’ perceptions of people with disability and their health care. Health Aff (Millwood). 2021;40(2):297-306. doi:10.1377/hlthaff.2020.01452 33523739 PMC8722582

[3] National Council on Disability. Framework to End Health Disparities of People with Disabilities. August 2022. Accessed November 9, 2025. https://www.ncd.gov/assets/uploads/reports/2022/ncd_health_equity_framework.pdf

[4] Havercamp SM, Scott HM. National health surveillance of adults with disabilities, adults with intellectual and developmental disabilities, and adults with no disabilities. Disabil Health J. 2015;8(2):165-172. doi:10.1016/j.dhjo.2014.11.002 25595297

[5] Paul S, Rafal M, Houtenville A. Annual Disability Statistics Compendium. University of New Hampshire Institute on Disability. 2020. Accessed November 9, 2025. https://files.eric.ed.gov/fulltext/ED613086.pdf

[6] Reichard A, Stolzle H, Fox MH. Health disparities among adults with physical disabilities or cognitive limitations compared to individuals with no disabilities in the United States. Disabil Health J. 2011;4(2):59-67. doi:10.1016/j.dhjo.2010.05.003 21419369

[7] Prusynski RA, Leland NE, Humbert A, . Post-acute care admissions among Medicare beneficiaries with disabilities during payment reform and the COVID-19 pandemic. Gerontologist. 2025;65(6):gnae180. doi:10.1093/geront/gnae180 39679946 PMC12082293

[8] Mathews M, Agniel D, Elliott MN, . The development of a patient-reported functional limitations index. Am J Manag Care. 2020;26(7):e225-e231. doi:10.37765/ajmc.2020.43765 32672921

[9] Iezzoni LI, Wint AJ, Tishler L, Palmisano J, Tripodis Y. Using claims for long-term services and support to predict mortality and hospital use. Disabil Health J. 2019;12(3):523-527. doi:10.1016/j.dhjo.2019.03.002 30956088

[10] Palsbo SE, Sutton CD, Mastal MF, Johnson S, Cohen A. Identifying and classifying people with disabilities using claims data: further development of the access risk classification system (ARCS) algorithm. Disabil Health J. 2008;1(4):215-223. doi:10.1016/j.dhjo.2008.07.001 21122732

[11] Ben-Shalom Y, Stapleton DC. Predicting disability among community-dwelling Medicare beneficiaries using claims-based indicators. Health Serv Res. 2016;51(1):262-281. doi:10.1111/1475-6773.12316 26015332 PMC4722209

[12] Kronick R, Gilmer T, Dreyfus T, Lee L. Improving health-based payment for Medicaid beneficiaries: CDPS. Health Care Financ Rev. 2000;21(3):29-64. 11481767 PMC4194678

[13] Office of the Inspector General. The Social Security Administration’s Expansion of Health Information Technology to Obtain and Analyze Medical Records for Disability Claims. January 2022. Accessed November 9, 2025. https://oig.ssa.gov/assets/uploads/a-01-18-50342summary.pdf

[14] Centers for Medicare & Medicaid Services. Original Medicare CAHPS. CMS.gov. Accessed December 16, 2025. https://www.cms.gov/data-research/research/consumer-assessment-healthcare-providers-systems/fee-service-cahps

[15] CAHPS Survey National Response Rates. July 30, 2024. Accessed December 16, 2025. https://ma-pdpcahps.org/globalassets/ma-pdp/comparative-data/2024/current-and-historic-cahps-overall-response-rates.pdf

[16] Centers for Medicare & Medicaid Services. Integrated Data Repository (IDR). Accessed December 16, 2025. https://www.cms.gov/about-cms/information-systems/idr

[17] Health Services Advisory Group. MA & PDP CAHPS Individual-Level Weight Construction. August 2023. Accessed December 16, 2025. https://www.ma-pdpcahps.org/globalassets/ma-pdp/other-analytic-information/mapdp_individual_weighting.pdf

[18] Centers for Medicare & Medicaid Services. Data from: Chronic Conditions Data Warehouse. 2023. Baltimore, MD.

[19] Centers for Medicare & Medicaid Services. Risk Adjustment. Accessed December 16, 2025. https://www.cms.gov/medicare/payment/medicare-advantage-rates-statistics/risk-adjustment

[20] Charlson ME, Pompei P, Ales KL, MacKenzie CR. A new method of classifying prognostic comorbidity in longitudinal studies: development and validation. J Chronic Dis. 1987;40(5):373-383. doi:10.1016/0021-9681(87)90171-8 3558716

[21] Gilmer T, Kronick R, Fishman P, Ganiats TG. The Medicaid Rx model: pharmacy-based risk adjustment for public programs. Med Care. 2001;39(11):1188-1202. doi:10.1097/00005650-200111000-00006 11606873

[22] Winters BD, Bharmal A, Wilson RF, . Validity of the Agency for Health Care Research and Quality Patient Safety Indicators and the Centers for Medicare and Medicaid Hospital-acquired Conditions: a systematic review and meta-analysis. Med Care. 2016;54(12):1105-1111. doi:10.1097/MLR.0000000000000550 27116111

[23] World Health Organization. Target Product Profiles for Priority Diagnostics to Support Response to the COVID-19 Pandemic. Version 1.0. Geneva, Switzerland: World Health Organization; 2020. https://www.who.int/publications/m/item/covid-19-target-product-profiles-for-priority-diagnostics-to-support-response-to-the-covid-19-pandemic-v.0.1

[24] Hays RD, Haas A, Haviland AM, . Factors associated with self-reports of limitations in activities of daily living among Medicare fee-for-service recipients. BMC Geriatr. 2024;24(1):648. doi:10.1186/s12877-024-05242-4 39090545 PMC11295787

[25] Jindai K, Nielson CM, Vorderstrasse BA, Quiñones AR. Multimorbidity and functional limitations among adults 65 or older, NHANES 2005-2012. Prev Chronic Dis. 2016;13:E151. doi:10.5888/pcd13.160174 27809419 PMC5094859

[26] Park S, Stimpson JP, Fendrick AM. Health care utilization patterns among adults with or without functional disabilities. JAMA Netw Open. 2025;8(4):e254729. doi:10.1001/jamanetworkopen.2025.4729 40214987 PMC11992608

[27] Xie Z, Hong YR, Tanner R, Marlow NM. People with functional disability and access to health care during the COVID-19 pandemic: a US population-based study. Med Care. 2023;61(2):58-66. doi:10.1097/MLR.0000000000001765 36040096 PMC9830960

[28] Klein DJ, Elliott MN, Haviland AM, . Understanding nonresponse to the 2007 Medicare CAHPS survey. Gerontologist. 2011;51(6):843-855. doi:10.1093/geront/gnr046 21700769

