# Supplementary material for: Use of Claims Data to Screen for Functional Limitations Among Medicare Beneficiaries
Source: JAMA Health Forum. 2026 Jun 12;7(6):e261388. doi: 10.1001/jamahealthforum.2026.1388 (PMC13263777; doi:10.1001/jamahealthforum.2026.1388)
Supplement: Supplement 1. — eMethods. Inclusion Criteria for Validation Sample eTable 1. Access Risk Classification System and Chronic Conditions Warehouse Claims-Based Indicators of Functional Status eTable 2. Chronic Illness and Disability Payment System (CDPS) Claims-Based Indicators of Functional Status eTable 3. Claims-Based Indicators of Functional Status and Indices Not in the Access Risk Classification System, Chronic Conditions Warehouse, or Chronic Illness and Disability Payment System eTable 4. Weighted Endorsement Rates of (Instrumental) Activities of Daily Living Among Eligible 2024 FFS CAHPS Survey Respondents (N = 63 535) eTable 5. Survey-Based FCFLI Linear Regression Model Coefficients eTable 6. Overall Means and Confidence Intervals for Binary Claims-Based Indicators of Functional Status for the Full Medicare Fee-for-Service Population and FCFLI-Claims Analytic Set eTable 7. Continuous Claims-based Indicators of Functional Status, Overall Means and Confidence Intervals for the Full Medicare Fee-for-Service Population and FCFLI-Claims Analytic Set eTable 8. Linear Model Results Predicting 2024 Survey-Based FCFLI Scores from Claims Variables (1-Year Lookback) eTable 9. Best Cut Points for Creating Functioning Groups Based on Positive Predicted Value (PPV) eTable 10. Distribution of 2024 Fee-for-Service Medicare Enrollees At Least Somewhat Functionally Limited by the FCFLI-Claims (score≤−1.00) Demographic Characteristics by Disability Insurance Status Original Reason for Medicare Entitlement eFigure 1. Positive Predictive Value (PPV) and Limitation Group Cut Point Optimization [file jamahealthforum-e261388-s001.pdf]

Supplemental Online Content

Mathews M, Martino SC, Agniel D, et al. Use of claims data to screen for functional limitations among Medicare beneficiaries. *JAMA Health Forum.* 2026;78(6):e261388. doi:10.1001/jamahealthforum.2026.1388

eMethods. Inclusion Criteria for Validation Sample
eTable 1. Access Risk Classification System and Chronic Conditions Warehouse Claims-Based Indicators of Functional Status
eTable 2. Chronic Illness and Disability Payment System (CDPS) Claims-Based Indicators of Functional Status
eTable 3. Claims-Based Indicators of Functional Status and Indices Not in the Access Risk Classification System, Chronic Conditions Warehouse, or Chronic Illness and Disability Payment System
eTable 4. Weighted Endorsement Rates of (Instrumental) Activities of Daily Living Among Eligible 2024 FFS CAHPS Survey Respondents (N = 63,535)
eTable 5. Survey-Based FCFLI Linear Regression Model Coefficients
eTable 6. Overall Means and Confidence Intervals for Binary Claims-Based Indicators of Functional Status for the Full Medicare Fee-for-Service Population and FCFLI-Claims Analytic Set
eTable 7. Continuous Claims-based Indicators of Functional Status, Overall Means and Confidence Intervals for the Full Medicare Fee-for-Service Population and FCFLI-Claims Analytic Set
eTable 8. Linear Model Results Predicting 2024 Survey-Based FCFLI Scores from Claims Variables (1-Year Lookback)
eTable 9. Best Cut Points for Creating Functioning Groups Based on Positive Predicted Value (PPV)
eTable 10. Distribution of 2024 Fee-for-Service Medicare Enrollees At Least Somewhat Functionally Limited by the FCFLI-Claims (score ≤ -1.00) Demographic Characteristics by Disability Insurance Status Original Reason for Medicare Entitlement
eFigure 1. Positive Predictive Value (PPV) and Limitation Group Cut Point Optimization

This supplemental material has been provided by the authors to give readers additional information about their work.

© 2026 Mathews M et al. *JAMA Health Forum*.

eMethods

Inclusion Criteria for Validation Sample

To validate the FCFLI-Claims prediction model, we used data from 82,337 adult respondents to the 2023 FFS CAHPS survey who completed both the ADL and self-rated health items. We excluded 3,070 respondents who did not answer all seven ADL items or the self-rated health question, and 6,526 respondents who were not continuously enrolled in both Medicare Parts A and B between April 2022 and March 2023. The final validation sample included 72,741 respondents from the 2023 FFS CAHPS survey.

Additional Detail on the Development of the FCFLI

To construct the FCFLI, we tested alternative ways of coding and modeling the seven functional limitation items from the FFS CAHPS survey. Six items assessed difficulty or inability in bathing, dressing, eating, transferring (getting in and out of a chair), walking, and toileting; the seventh item assessed difficulty running errands alone.

Coding of Functional Limitation Items

For the six multi-level items, we compared two parameterizations: (1) binary indicators for each severity level (“difficulty” and “unable,” with “no difficulty” as the reference) and (2) linear scoring of severity (0 = no difficulty, 1 = difficulty, 2 = unable). The running-errands-alone item was coded as a binary indicator (0 = no difficulty, 1 = difficulty).

To capture the cumulative effect of multiple limitations, we also created two count variables: the number of ADLs beyond one with difficulty (maximum = 6) and the number beyond one with inability (maximum = 5).

Modeling Approaches Considered

We evaluated two regression approaches for predicting self-rated health from these limitation measures: (1) ordinary least squares (OLS) linear regression including main effects for each item and (2) elastic-net regularized regression allowing for interactions among items and penalizing overfitting. Both models included the same covariates (age, education, LIS status, and census division). Model performance was assessed using root mean squared error (RMSE) for both within-sample (2024 CAHPS) and out-of-sample (2023 CAHPS) predictions.

Root mean square error (RMSE) was calculated to assess prediction error using the following formula to account for weights:

© 2026 Mathews M et al. *JAMA Health Forum*.

$$\sqrt{\frac{\sum_{i=1}^{N_s}(W_i * e_i)^2}{\sum_{i=1}^{N_s}(W_i)}}$$

where W_i is the FFS CAHPS respondent weight and e_i is the predicted residual value for each *i* respondent in each *s* population of interest, ie, those included in either the 1-year (N = 57 544) or 2-year (N = 72 741) claims lookback period). Each model predicted FCFLI scores, standardized with mean 0 and standard deviation 1, from a set of claims indicators.

Prediction errors were nearly identical between the two approaches (within-sample RMSE ≈ 20.8; out-of-sample ≈ 21.0). Because the elastic-net model offered no improvement in accuracy and was less interpretable, we selected the simpler linear regression model for constructing the FCFLI.

Final Coding Decision

We then compared the linear regression model using binary indicators with a version using linearly scored ADLs. The linearly scored model assumed equal intervals between severity levels and produced slightly higher RMSEs (within-sample 20.98 vs. 20.88; out-of-sample 21.09 vs. 20.98) but provided a clearer representation of incremental severity effects. In nearly all ADLs, the negative association with self-rated health increased from “difficulty” to “unable,” supporting the linear scoring assumption.

Based on these results, we adopted the linearly scored ADL specification for the final FCFLI, balancing interpretability, parsimony, and conceptual coherence.

Additional Detail on Random Forest Modeling Approach to Predicting FCFLI Scores

A random forest modeling approach was implemented using the ranger package in R (v4.4.1). The model constructed an ensemble of decision trees, each trained on a bootstrap sample of the 2024 dataset, with random feature selection at each split to reduce correlation among trees. The final prediction was obtained by majority voting across all trees.

Model hyperparameters were optimized using a grid search combined with 5-fold cross-validation on the training set, implemented using the caret package in R. The parameters tuned included:

- Number of variables tried at each split (*mtry*): $(\sqrt{p})*2$, $p/10$, $p/6$, $p/4$, $\sqrt{p}$, $(\sqrt{p})/2$, $p/3$, where p represents the number of predictors (p=43)
- Minimum node size (*min.node.size*): 5, 10, 20, 50, 150, 300.
- Minimum number of trees (*n.trees*): 500

© 2026 Mathews M et al. *JAMA Health Forum*.

Each tree in the random forest was trained on a random subsample (without replacement) comprising 60% of the 2024 training data (*sample.fraction* = 0.6). This approach reduces correlation among trees and helps prevent overfitting by increasing ensemble diversity.

Unordered categorical variables were split using partitioning (*respect.unordered.factors* = “partition”) rather than imposing an arbitrary numeric ordering, to improve model accuracy. The optimal hyperparameter combination was selected based on the lowest mean cross-validated mean squared error.

Variable importance was assessed using the permutation-based mean decrease in accuracy (*importance* = “permutation”).

© 2026 Mathews M et al. *JAMA Health Forum*.

eTable 1. Access Risk Classification System and Chronic Conditions Warehouse Claims-Based Indicators of Functional Status

Indicator	Description
Access Risk Classification System (ARCS) indicators	Classifies people into one of four binary categories based on the probable need for care coordination or health system accommodations.
No risk (level=0)	Emergent or acute medical needs (e.g., pregnancy, any emergent condition)
Low risk (level=1)	Fewer than 2 chronic conditions that cause some functional limitations (e.g., uses no or simple compensatory devices, like a walker; uncontrolled diabetes; early stages of multiple sclerosis)
Medium risk (level=2)	1 or more chronic conditions that cause major functional limitations (e.g., bipolar disorder; minor intellectual disability; recent spinal cord injury)
High risk (level=3)	Multiple chronic conditions and complex medical needs that severely impair a person's independence (e.g., spinal cord injury with traumatic brain injury and diabetes; advanced stages of multiple sclerosis)
Medicare Chronic Conditions Warehouse (CCW) condition indicators	Identifies the prevalence of chronic conditions through diagnosis and procedure claims codes. While 64 main and supplemental chronic conditions are available, we only retained 10 binary chronic condition categories that had non-overlapping information with our other claims-based disability indicators.
Acquired hypothyroidism	These four chronic condition variables are developed from algorithms that search Medicare administrative claims data for specific ICD-10, MS-DRG codes, or HCPCS codes.
Anemia	
Glaucoma	
Stroke/transient ischemic attack	
Migraine and chronic headache	These six chronic conditions are considered supplemental or rare conditions and are not covered by any category outlined in other algorithms. ICD-10 codes were used to create these indicators. We used phenome-wide association study (PheWAS) categories to identify these ICD-10 codes, as the available linkable CCW data contained only common conditions.
Mobility impairments	
Obesity	
Blindness and visual impairment	
Deafness and hearing impairment	
Tobacco use	

Notes. MS-DRG = Medicare Severity Diagnosis Related Groups; ICD-10 = International Classification of Diseases version 10; HCPCS = Healthcare Common Procedure Coding System. All MS-DRG, ICD-10, and HCPCS codes from the following Medicare claims files were utilized to create our claims-based disability indicators: Master Beneficiary Summary Segment files on Chronic Conditions and Cost and Utilization, Medicare Provider and Analysis Review, Carrier Claims, Durable Medical Equipment, Home Health Agency Claims and Revenue, Hospice Claims and Revenue, and Outpatient Claims and Revenue. We used preexisting software or SAS codes presented in technical documentation of the original development work for each indicator to assign each respondent per year values from the claims-based indicators of functional status.

© 2026 Mathews M et al. *JAMA Health Forum*.

eTable 2. Chronic Illness and Disability Payment System (CDPS) Claims-Based Indicators of Functional Status

<table>
<tr><th>Indicator</th><th>Description</th></tr>
<tr><td>Chronic Illness and Disability Payment System (CDPS) indicators</td><td>The CDPS is a diagnosis-based risk adjustment model used by Medicaid programs to adjust capitated payments to managed care organizations for beneficiaries with chronic illnesses and disabilities. It groups ICD-coded diagnoses into categories representing major body systems or chronic disease types. Each category has a hierarchy reflecting clinical severity and expected future costs and is assigned a weight. These weights are summed across categories to generate a total risk score.</td></tr>
<tr><td>Acquired Immunodeficiency Syndrome (AIDS)</td><td rowspan="22">We created linearized condition variables by assigning the following values to cost burden levels: 0=no cost, 1=extra low, 2=very low, 3=low, 4=medium low, 5=medium, 6=high, 7=very high, 8=extra high cost. All categories were created by grouping various ICD-10 codes.

Lack of observed illness burden in our analytic data for some of the original 22 categories led us to combine AIDS and HIV indicators and drop substance abuse and heart and lung transplant indicators, resulting in a final set of 18 categories</td></tr>
<tr><td>Cancer</td></tr>
<tr><td>Cardiovascular</td></tr>
<tr><td>Central nervous system disorders</td></tr>
<tr><td>Developmental disability, e.g., intellectual disability, cerebral palsy, epilepsy, autism spectrum disorder</td></tr>
<tr><td>Diabetes, type 1</td></tr>
<tr><td>Diabetes, type 2</td></tr>
<tr><td>Eye</td></tr>
<tr><td>Genital</td></tr>
<tr><td>Gastrointestinal</td></tr>
<tr><td>Hematological</td></tr>
<tr><td>Human Immunodeficiency Virus (HIV)</td></tr>
<tr><td>Heart and lung transplant status</td></tr>
<tr><td>Infectious</td></tr>
<tr><td>Metabolic</td></tr>
<tr><td>Psychiatric</td></tr>
<tr><td>Pulmonary</td></tr>
<tr><td>Renal</td></tr>
<tr><td>Skeletal</td></tr>
<tr><td>Skin</td></tr>
<tr><td>Substance abuse</td></tr>
<tr><td>No CDPS category (reference)</td></tr>
</table>

Notes. ICD-10 = International Classification of Diseases version 10. ICD-10 codes from the following Medicare claims files were utilized to create our claims-based disability indicators: Master Beneficiary Summary Segment files on Chronic Conditions and Cost and Utilization, Medicare Provider and Analysis Review, Carrier Claims, Durable Medical Equipment, Home Health Agency Claims and Revenue, Hospice Claims and Revenue, and Outpatient Claims and Revenue. We used preexisting software or SAS codes presented in technical documentation of the original development work for each indicator to assign each respondent per year values from the claims-based indicators of functional status.

© 2026 Mathews M et al. *JAMA Health Forum*.

eTable 3. Claims-Based Indicators of Functional Status and Indices Not in the Access Risk Classification System, Chronic Conditions Warehouse, or Chronic Illness and Disability Payment System

Indicator	Description
Social Security Administration - Health Information Technology (SSA-HIT) indicator	The SSA-HIT classifies individuals as either disabled or not disabled based on SSA business rules for determining disability benefit eligibility. Conditions triggering classification as disabled include a wide range of severe or chronic diagnoses (e.g., amyotrophic lateral sclerosis, cerebral palsy, end-stage renal disease, spinal cord injury, traumatic brain injury, intellectual disability, and major chromosomal disorders such as trisomy 13, 18, and 21). The list also includes advanced-stage cancers (e.g., of the lung, liver, pancreas, breast, prostate, and hematologic malignancies such as lymphoma and leukemia), major organ transplants within the past 12 months (lung, heart, liver, kidney, bone marrow, or stem cell), and other serious conditions (e.g., blindness, amputation of both hands, persistent vegetative state, and schizophrenia).<br>ICD-10 codes are used to identify these conditions.
Hierarchical Condition Category (HCC) risk adjuster	The HCC risk adjustor is a statistical tool used by Medicare and other insurers to estimate a patient's expected healthcare costs based on their health status, demographics, and diagnoses. It assigns a risk adjustment score to each individual, which reflects the likelihood of higher-than-average medical spending. A risk adjustment score above 1 indicates that the patient is expected to cost more than average, and a score below 1 indicates that the patient is expected to cost less than average. HCC relies on ICD-10 codes to assign risk scores for patients. HCPCS codes are used to determine whether a service is included in the calculation. We used the relevant HCC model to calculate the index score for each year of data, e.g., 2024 CMS-HCC Model Software was used to calculate the HCC scores for the 2024 FFS data.
Quan-Charlson Comorbidity Index	The Quan-Charlson Comorbidity Index is a continuous measure of comorbidity in which a score of zero indicates no c comorbid conditions and higher scores reflect greater predicted risk of mortality or resource use. Seventeen comorbidity categories, identified using ICD-10 codes, are each assigned a weight from 1 to 6 based on their adjusted association with mortality or resource utilization.
Medicaid Rx Model	The Medicaid Rx model is a continuous index that estimates the cost burden of an individual's comorbidities based on 45 weighted prescription drug categories. These categories are created by grouping medications according to their National Drug Codes.
Healthcare utilization indicators	These indicators include total annual utilization days across a variety of services, including acute inpatient, other inpatient, skilled nursing facility, and hospice patients.
Hospital readmissions	

Notes. ICD-10 = International Classification of Diseases version 10. HCPCS = Healthcare Common Procedure Coding System. All ICD-10 and NDC codes from the following Medicare claims files were utilized to create our claims-based disability indicators: Master Beneficiary Summary Segment files on Chronic Conditions and Cost and Utilization, Medicare Provider and Analysis Review, Carrier Claims, Durable Medical Equipment, Home Health Agency Claims and Revenue, Hospice Claims and Revenue, Outpatient Claims and Revenue, and Medicare Part D Drug Event. We used preexisting software or SAS codes presented in technical documentation of the original development work for each indicator to assign each respondent per year values from the claims-based indicators of functional status.

© 2026 Mathews M et al. *JAMA Health Forum*.

eTable 4. Weighted Endorsement Rates of (Instrumental) Activities of Daily Living Among Eligible 2024 FFS CAHPS Survey Respondents (N = 63,535)

Severity	Bathing	Dressing	Eating	Getting in and out of a chair	Walking	Using the toilet	Running errands alone
**0: No difficulty**	87.0%	88.6%	93.9%	82.5%	75.1%	91.3%	84.4%
**1: Has difficulty**	8.7%	7.6%	2.8%	13.9%	20.8%	5.1%	15.6%
**2: Unable to do**	4.3%	3.8%	3.3%	3.6%	4.1%	3.6%	—

© 2026 Mathews M et al. *JAMA Health Forum*.

eTable 5. Survey-Based FCFLI Linear Regression Model Coefficients

**Effect**	**Estimate**	**95% Confidence Interval**
Intercept	61.4	60.8, 62.0
I/ADL per unit of limitation (0= no difficulty, 1= has difficulty, 2= unable to do)		
Bathing	-6.3	-7.5, -5.1
Dressing	-6.0	-7.4, -4.5
Eating	-6.7	-8.1, -5.2
Getting in and out of a chair	-9.0	-10.0, -8.0
Walking	-13.9	-14.6, -13.2
Using the toilet	-3.3	-4.8, -1.8
Difficulty running errands alone (0= no difficulty, 1= has difficulty)	-11.7	-12.7, -10.8
Count of limitations beyond 1, by severity level		
Have difficulty (per 0-6 additional limitations)	3.9	3.1, 4.7
Unable to do (per 0-5 additional limitations)	16.6	14.9, 18.2
LIS Indicator	-6.1	-8.4, -3.7
Age [reference: *70-74*]		
18 - 64	-5.6	-6.8, -4.5
65 - 69	0.8	0.2, 1.4
75 - 79	-1.2	-1.7, -0.7
80 - 84	-1.3	-1.9, -0.7
85 and older	0.2	-0.4, 0.9
Educational attainment [reference: *some college*]		
Less than high school	-7.1	-8.9, -5.4
Some high school	-5.3	-6.5, -4.0
High school graduate / GED	-2.5	-3.0, -1.9
Bachelor's degree	4.0	3.4, 4.6
Advanced college degree	6.3	5.8, 6.9
Census Division [reference: *South Atlantic*]		
New England	0.5	-0.3, 1.4
Mid Atlantic	-0.5	-1.3, 0.2
East South Central	-2.8	-3.7, -1.9
East North Central	-0.4	-1.1, 0.3
West South Central	-1.1	-1.8, -0.3
West North Central	0.3	-0.4, 1.1
Mountain	0.1	-0.7, 0.9
Pacific	-0.8	-1.5, -0.1
Missing	-5.5	-7.2, -3.8

Notes: Weighted linear regression predicting 0-100 scored self-reported health status among 63,535 respondents.
Adjusted R^2 for the model: 0.257371
Root mean square error: 20.98

© 2026 Mathews M et al. *JAMA Health Forum*.

eTable 6. Overall Means and Confidence Intervals for Binary Claims-Based Indicators of Functional Status for the Full Medicare Fee-for-Service Population and FCFLI-Claims Analytic Set

**Claims-based indicator of functional status**	**All Medicare FFS enrollees** (N = 31,539,848)		**Included in FCFLI-Claims Analysis*** (*N* = 57,544)	
	Mean	95% Confidence Interval	Mean	95% Confidence Interval
ARCS[a]				
No risk	10.1%	10.1%, 10.1%	4.7%	4.5%, 4.9%
Low risk	3.6%	3.6%, 3.6%	3.0%	2.9%, 3.1%
Medium risk	14.2%	14.2%, 14.2%	14.8%	14.5%, 15.1%
High risk	72.0%	72.0%, 72.1%	77.5%	77.1%, 77.8%
CCW[a]				
Glaucoma	11.8%	11.8%, 11.8%	14.8%	14.6%, 15.1%
Stroke/transient ischemic attack	6.0%	5.9%, 6.0%	4.9%	4.7%, 5.1%
Anemia	15.0%	15.0%, 15.0%	13.8%	13.5%, 14%
Acquired hypothyroidism	15.7%	15.7%, 15.7%	17.2%	16.9%, 17.5%
Migraine and chronic headache	2.3%	2.3%, 2.3%	2.5%	2.4%, 2.6%
Mobility impairment	2.0%	2.0%, 2.0%	1.2%	1.1%, 1.3%
Obesity	13.6%	13.6%, 13.6%	15.0%	14.7%, 15.3%
Blindness and visual impairment	0.3%	0.3%, 0.3%	0.1%	0.1%, 0.2%
Deafness and hearing impairment	3.7%	3.7%, 3.7%	4.0%	3.9%, 4.2%
Tobacco use	8.0%	8.0%, 8.0%	6.8%	6.6%, 7.0%
SSA-HIT indicator[a]	39.3%	39.3%, 39.4%	40.0%	39.6%, 40.4%

Notes. * Those included in the FCFLI-Claims analysis responded to the 2024 FFS CAHPS survey and had linked claims data during the 1-year lookback period. Percentages shown were adjusted using FFS CAHPS respondent weights.

[a] These claim-based indicators have binary distributions (1/0).

FFS = Fee-for-Service

ARCS= Access Risk Classification System

CCW= Chronic Conditions Warehouse

SSA-HIT= Social Security Administration - Health Information Technology

© 2026 Mathews M et al. *JAMA Health Forum*.

eTable 7. Continuous Claims-based Indicators of Functional Status, Overall Means and Confidence Intervals for the Full Medicare Fee-for-Service Population and FCFLI-Claims Analytic Set

**Claims-based indicator of functional status**	**All Medicare FFS enrollees** (N = 31,539,848)		**Included in FCFLI-Claims Analysis*** (*N* = 57,544)	
	Mean	95% Confidence Interval	Mean	95% Confidence Interval
CDPS[a]				
No CDPS category	0.13	0.13, 0.13	0.06	0.06, 0.07
AIDS or HIV	0.05	0.05, 0.05	0.04	0.03, 0.04
Cancer	1.30	1.30, 1.30	1.52	1.50, 1.54
Cardiovascular	1.93	1.93, 1.93	2.00	1.99, 2.02
Central nervous system disorders	1.58	1.58, 1.58	1.76	1.75, 1.77
Developmental disabilities	0.09	0.09, 0.09	0.06	0.05, 0.06
Diabetes, type 1	0.03	0.03, 0.03	0.02	0.02, 0.02
Diabetes, type 2	0.07	0.07, 0.07	0.06	0.05, 0.06
Eye	0.97	0.97, 0.97	0.98	0.96, 0.99
Genital	0.76	0.76, 0.76	0.93	0.92, 0.94
Gastrointestinal	0.23	0.23, 0.23	0.23	0.23, 0.23
Hematological	1.13	1.12, 1.13	1.17	1.15, 1.18
Infectious	0.27	0.27, 0.27	0.28	0.27, 0.29
Metabolic	0.25	0.25, 0.25	0.19	0.19, 0.20
Psychiatric	0.82	0.82, 0.82	0.77	0.76, 0.79
Pulmonary	0.87	0.87, 0.87	0.72	0.71, 0.73
Renal	0.97	0.96, 0.97	0.87	0.85, 0.88
Skeletal	1.06	1.06, 1.06	1.05	1.03, 1.07
Skin	1.29	1.29, 1.29	1.44	1.42, 1.45
Substance Abuse	0.37	0.37, 0.38	0.31	0.30, 0.32
Quan-Charlson comorbidity index[a]	0.11	0.11, 0.11	0.09	0.08, 0.09
Medicaid Rx model[b]	1.62	1.62, 1.62	1.55	1.54, 1.57
HCC risk adjustor[b]	1.51	1.51, 1.51	1.36	1.35, 1.37
Healthcare utilization[a]	0.99	0.99, 1.00	0.92	0.91, 0.92
Covered care days	5.07	5.06, 5.08	2.11	1.98, 2.24
Hospital readmissions	0.04	0.04, 0.04	0.02	0.02, 0.02

Notes. * Those included in the FCFLI-Claims analysis responded to the 2024 FFS CAHPS survey and had linked claims data during the 1-year lookback period. Percentages shown were adjusted using FFS CAHPS respondent weights.

a These claims-based indicators have count distributions starting at zero and represent severity or days. CDPS categories ranges from 0 to 8. Quan-Charlson comorbidity index ranges from 0 to 17.

b These claims-based indicators are continuous.

CDPS= Chronic Illness and Disability Payment System

FCFLI = Fee-for-Service CAHPS Functional Limitation Index

FFS = Fee-for-Service

HCC = Hierarchical Conditions Category

Rx = prescription

© 2026 Mathews M et al. *JAMA Health Forum*.

eTable 8. Linear Model Results Predicting 2024 Survey-Based FCFLI Scores from Claims Variables (1-Year Lookback)

Effect	Estimate	95% Confidence Interval
Intercept	0.594	0.443, 0.745
Healthcare utilization[b]		
Covered care days	-0.004	-0.005, -0.003
Readmissions	0.069	0.004, 0.134
Medicaid Rx risk[c]	0.010	0.002, 0.018
Quan-Charlson Comorbidity Index[b]	-0.023	-0.036, -0.010
SSA-HIT indicator[a]	0.040	0.014, 0.066
HCC risk adjustor[c]	-0.267	-0.290, -0.244
ARCS[a]		
Low risk	0.092	0.016, 0.168
Medium risk	0.193	0.133, 0.253
High risk	0.189	0.131, 0.248
CCW[a]		
Anemia	-0.046	-0.079, -0.013
Blindness/visual impairment	-0.543	-0.911, -0.175
Deafness	-0.051	-0.100, -0.002
Glaucoma	-0.013	-0.038, 0.012
Hypothyroidism	0.002	-0.023, 0.027
Migraine	-0.098	-0.167, -0.029
Mobility	-0.433	-0.600, -0.265
Obesity	-0.147	-0.178, -0.117
Stroke/transient ischemic attack	-0.068	-0.122, -0.013
Tobacco	-0.085	-0.128, -0.042
CDPS[b]		
Central nervous system	-0.006	-0.013, 0.000
Cerebrovascular	-0.111	-0.151, -0.071
Developmental disability	-0.163	-0.217, -0.109
Diabetes, Type 1	-0.004	-0.024, 0.016
Diabetes, Type 2	-0.017	-0.025, -0.009
Eye	0.066	0.057, 0.075
Gastrointestinal	0.014	0.007, 0.020
Genital	-0.072	-0.097, -0.048
Hematological	0.029	0.018, 0.040
Infectious	0.010	-0.004, 0.023
Metabolic	0.006	0.000, 0.012
Psychiatric	-0.047	-0.054, -0.039
Pulmonary	-0.010	-0.017, -0.003
Renal	-0.008	-0.014, -0.003
Skeletal	-0.039	-0.045, -0.033
Skin	-0.072	-0.085, -0.058
Substance abuse	-0.046	-0.072, -0.021
Aids / HIV	0.027	0.002, 0.052
Cancer	0.009	0.005, 0.014
Cardiovascular	-0.014	-0.021, -0.007
Age[c]	-0.004	-0.005, -0.002
DE/LIS[a]	-0.621	-0.670, -0.571
Male[a]	0.057	0.036, 0.079
Institutionalized[a]	-0.423	-0.620, -0.225
Number of months enrolled in FFS[b]	-0.014	-0.022, -0.005
Continuous Part D enrollment[a]	0.125	0.103, 0.146

© 2026 Mathews M et al. *JAMA Health Forum*.

Notes. Weighted linear regression predicting standardized (mean=0, standard deviation=1) survey-based FCFLI scores among 57,544 FFS CAHPS survey respondents who had 2024 claims data. All results are weighted using MA & PDP individual weights.
R^2 for the model: 0.2418
Root mean square error: 0.9103
[a] These claim-based indicators have binary distributions (1/0).
[b] These claims-based indicators have count distributions starting at zero and represent severity or days. CDPS categories ranges from 0 to 8. Quan-Charlson comorbidity index ranges from 0 to 17.
[c] These claims-based indicators are continuous.
ARCS= Access Risk Classification System
CCW= Chronic Conditions Warehouse
CDPS= Chronic Illness and Disability Payment System
FCFLI = Fee-for-Service CAHPS Functional Limitation Index
FFS = Fee-for-Service
HCC= Hierarchical Conditions Category
Rx=prescription
SSA-HIT= Social Security Administration - Health Information Technology

© 2026 Mathews M et al. *JAMA Health Forum*.

eTable 9. Best Cut Points for Creating Functioning Groups Based on Positive Predicted Value (PPV)

Option	Cut Points		Positive Predicted Values (PPV)			
	Least-Somewhat Limited	Somewhat-Most Limited	Least Limited	Somewhat Limited	Most Limited	At Least Somewhat Limited
**1**	-1.8	-2.3	91.7	18.1	100.0	85.9
**2**	-0.55	-2.1	84.1	51.3	87.0	77.1
**3**	-0.3	-2	86.3	43.7	93.1	70.0
**4**	-0.55	-2	84.1	43.7	93.1	77.1
**5**	-0.5	-2	84.7	43.5	93.1	75.5
**6**	-0.45	-2	85.4	43.4	93.1	73.8
**7**	-0.25	-2	86.9	43.4	93.1	68.3
**8**	-0.4	-2	86.0	43.2	93.1	72.1
**9**	-0.1	-2	87.2	43.1	93.1	64.4
**10**	-0.35	-2	86.7	42.7	93.1	70.3
**11**	-0.2	-2	87.7	42.6	93.1	66.3
**12**	-0.05	-2	87.8	42.4	93.1	62.6
**13**	0	-2	88.5	42.0	93.1	61.0
**14**	-0.15	-2	88.5	41.9	93.1	64.5
**15**	-0.65	-2	85.0	37.1	93.1	74.6
**16**	-0.7	-2	84.4	36.8	93.1	76.6
**17**	-0.6	-2	85.6	36.8	93.1	72.3
**18**	-0.75	-2	83.9	36.4	93.1	78.6
**19**	-0.8	-2	88.6	29.5	93.1	74.0
**20**	-0.85	-2	88.2	28.7	93.1	75.2
**21**	-0.9	-2	87.8	28.7	93.1	76.9
**22**	-0.95	-2	87.3	28.4	93.1	78.7
**23**	-1	-2	87.1	28.3	93.1	80.4
**24**	-1.05	-2	87.1	27.1	93.1	81.0
**25**	-1.1	-2	87.0	26.3	93.1	81.5
**26**	-1.15	-2	86.7	25.9	93.1	83.1
**27**	-1.2	-2	86.4	25.6	93.1	84.4
**28**	-1.25	-2	86.6	25.4	93.1	85.2
**29**	-1.3	-2	89.6	23.3	93.1	84.2
**30**	-1.45	-2	89.3	18.8	93.1	84.3
**31**	-1.35	-2	89.6	18.8	93.1	81.5
**32**	-1.4	-2	89.4	18.7	93.1	83.2
**33**	-1.5	-2	89.2	18.5	93.1	85.3
**34**	-1	-2	87.1	28.3	93.1	80.4
**35**	-1	-1.95	87.1	27.3	90.1	80.4
**36**	-1	-1.9	87.1	25.2	89.8	80.4
**37**	-1	-2.05	87.1	30.9	88.1	80.4
**38**	-1	-2.1	87.1	37.0	87.0	80.4
**39**	-1	-1.8	87.1	22.3	85.9	80.4
**40**	-1	-1.6	87.1	19.7	85.7	80.4
**41**	-1	-1.55	87.1	13.3	85.5	80.4
**42**	-1	-1.5	87.1	11.2	85.3	80.4
**43**	-1	-1.7	87.1	20.7	85.3	80.4
**44**	-1	-1.75	87.1	21.7	85.2	80.4
**45**	-1	-1.85	87.1	25.0	84.7	80.4
**46**	-1	-1.65	87.1	20.3	84.0	80.4

© 2026 Mathews M et al. *JAMA Health Forum*.

Notes. The “Somewhat-Most Limited” cut point was used only to distinguish the “Most Limited” group, while the “Least-Somewhat” cut point was used for grouping the “Somewhat Limited” group in the 3-level categorization as well as the “At Least Somewhat Limited” group in the binary categorization scheme. Not all cut point combinations for the 3-level categorization are shown in this table. We only show the most optimal PPV options for each level: option 1 optimizes on the “Least Limited” PPV, option 2 optimizes on the “Somewhat Limited” PPV, option 3-34 optimizes on the “Most Limited” PPV, and options 35-46 optimize on the “At Least Somewhat Limited” PPV.

© 2026 Mathews M et al. *JAMA Health Forum*.

eTable 10. Distribution of 2024 Fee-for-Service Medicare Enrollees At Least Somewhat Functionally Limited by the FCFLI-Claims (score ≤ -1.00) Demographic Characteristics by Disability Insurance Status Original Reason for Medicare Entitlement

Characteristic	Originally Entitled for Medicare Due to Age	Originally Entitled for Medicare Due to Disability
All Medicare FFS Enrollees At Least Somewhat Functionally Limited by the FCFLI-Claims (score ≤ -1.00)	62.9% (N=2,450,385)	37.1% (N=1,445,861)
Dual eligibility/LIS recipient status		
Either dually eligible or an LIS recipient	39.8%	68.9%
Neither dually eligible nor an LIS recipient	60.2%	31.1%
Age		
18-64	1.0%	55.5%
65-69	4.4%	12.7%
70-74	8.7%	10.9%
75-79	12.6%	8.7%
80-84	16.1%	6.0%
85+	57.2%	6.2%
Sex		
Male	37.2%	47.3%
Female	62.8%	52.7%

FCFLI = Fee-for-Service CAHPS Functional Limitation Index, FFS = Fee-for-Service, LIS = Low-Income Subsidy

© 2026 Mathews M et al. *JAMA Health Forum*.

eFigure 1.: Positive Predictive Value (PPV) and Limitation Group Cut Point Optimization

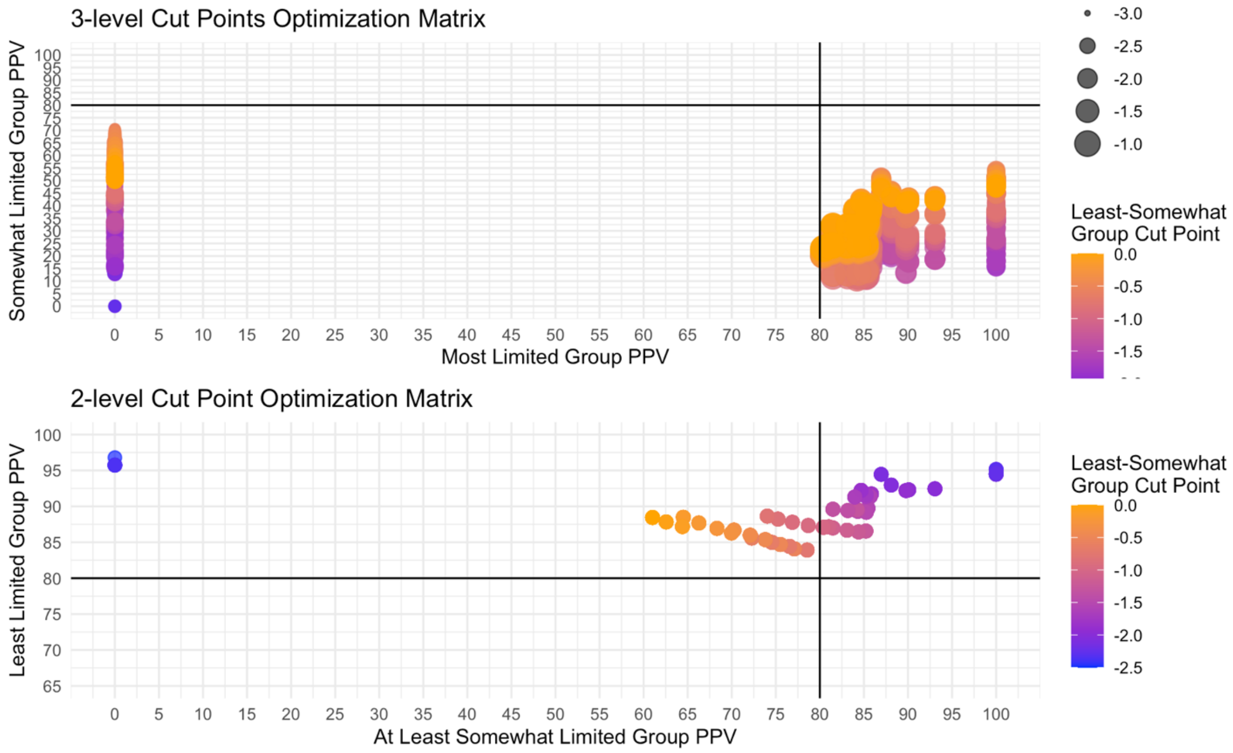

The top panel shows PPV values for the **most limited** group (x-axis) relative to PPV values for the **somewhat limited** group (y-axis) across all 1,269 three-category cut point combinations. The color of each point represents the *least-to-somewhat limited* cut point value, ranging from blue (–2.5) to orange (0), while point size represents the *somewhat-to-most limited* cut point value, ranging from smallest (–3.0) to largest (–1.0). The bottom panel shows PPV values for the **"at least somewhat limited"** group (x-axis) relative to PPV values for the **least limited** group (y-axis) across 51 two-category cut point options. The color of each point represents the *least-to-at-least-somewhat limited* cut point value, ranging from blue (–2.5) to orange (0). The dark vertical and horizontal lines at 80% PPV create a performance grid, with cut points positioned in the upper right quadrant considered optimal candidates.

© 2026 Mathews M et al. *JAMA Health Forum*.
