# Supplementary material for: Use of Claims Data to Screen for Functional Limitations Among Medicare Beneficiaries
Source: JAMA Health Forum. 2026 Jun 12;7(6):e261388. doi: 10.1001/jamahealthforum.2026.1388 (PMC13263777; doi:10.1001/jamahealthforum.2026.1388)
Supplement: Supplement 2. — Data Sharing Statement [file jamahealthforum-e261388-s002.pdf]

Data Sharing Statement

Mathews. Use of Claims Data to Screen for Functional Limitations Among Medicare Beneficiaries. *JAMA Health Forum*. Published June 12, 2026.
doi:10.1001/jamahealthforum.2026.1388

Data
Data available: No
